# The impact of the socioeconomic factor on Parkinson's disease medication adherence: a scoping review

**DOI:** 10.1055/s-0044-1779608

**Published:** 2024-02-23

**Authors:** Gustavo Gil, Michelle H. S. Tosin, Henrique Ballalai Ferraz

**Affiliations:** 1 Universidade Federal de São Paulo, Escola Paulista de Medicina, Departamento de Neurologia, São Paulo SP, Brazil.; 2 Rush University Medical Center, Department of Neurological Sciences, Chicago, Illinois, United States.

**Keywords:** Medication Adherence, Patient Compliance, Parkinson Disease, Socioeconomic Factors, Adesão à Medicação, Cooperação do Paciente, Tratamento da Doença de Parkinson, Fatores Socioeconômicos

## Abstract

**Background** Therapeutic adherence is a decisive issue on chronic disease management in patients requiring long-term pharmacotherapy, such as Parkinson's disease (PD). Although it is well known that socioeconomic factor is a barrier to medication adherence in many chronic diseases, its impacts on PD still need to be investigated.

**Objective** Explore what and how socioeconomic factors impact medication adherence in people with PD.

**Methods** We carried out a scoping review across three databases to identify studies exploring what and how socioeconomic factors impact medication adherence in people with PD considering eight attributes: 1. educational level, 2. disease-related knowledge, 3. income, 4. cost of medication, 5. drug subsidy (meaning presence of subsidies in the cost of medication), 6. employability, and 7. ethnicity (black, indigenous, immigrants).

**Results** Of the 399 identified studies (Embase = 294, Medline = 88, LILACS = 17), eight met inclusion criteria. We identified factors covering the eight attributes of socioeconomic impact, and all of them negatively impacted the medication adherence of people with PD. The most prevalent factor in the studies was low patient educational level (four studies), followed by costs of medications (three studies), income (three studies), and disease-related knowledge (three studies). Distinctly from most of the studies selected, one of them evidenced suboptimal adherence in individuals receiving the medication free of charge, and another one could not find correlation between suboptimal adherence and educational level.

**Conclusion** Socioeconomic factors negatively impact medication adherence in PD patients. This review provides basis for developing patient and population-based interventions to improve adherence to treatment in PD.

## Resumo

**Antecedentes** A adesão à medicação é um componente crucial no manejo correto da doença de Parkinson (DP) e, embora esteja bem estabelecido que o fator socioeconômico é uma barreira à adesão medicamentosa em muitas doenças crônicas, seus impactos na DP ainda precisam ser investigados.

**Objetivo** Explorar quais são e como os fatores socioeconômicos afetam a adesão à medicação em pessoas com DP.

**Métodos** Realizamos uma revisão de escopo em três bases de dados para identificar estudos que explorassem quais e como os fatores socioeconômicos impactam na adesão à medicação em pessoas com DP, considerando oito atributos: 1. nível educacional, 2. conhecimento relacionado à doença, 3. renda, 4. custo de medicamentos, 5. subsídio de medicamentos (ou seja, presença de subsídios no custo dos medicamentos), 6. empregabilidade e 7. etnia (negra, indígena, imigrantes).

**Resultados** Dos 399 estudos identificados (Embase = 294, Medline = 88, LILACS = 17), oito preencheram os critérios de inclusão. Identificamos fatores que abrangem os oito atributos de impacto socioeconômico e todos impactaram negativamente na adesão medicamentosa de pessoas com DP. Foram mais prevalentes o baixo nível educacional do paciente (quatro estudos), custos dos medicamentos, nível de renda e conhecimento relacionado à doença (três estudos cada). Diferentemente da maioria dos estudos selecionados, um deles evidenciou adesão subótima em indivíduos que receberam a medicação gratuitamente, e outro não encontrou correlação entre adesão subótima e nível educacional.

**Conclusão** Fatores socioeconômicos impactam negativamente a adesão ao tratamento medicamentoso em pessoas com DP. Esta revisão fornece base para o desenvolvimento de intervenções baseadas em pacientes e populações no intuito de melhorar a adesão ao tratamento farmacológico de pessoas com DP.

## INTRODUCTION

Parkinson's disease (PD) is a prevalent disease and affects approximately 4 million people. 1 The estimated incidence ranges from 5 to 35 new cases per 100,000 individuals per year, most affecting old-aged people, being more than 3% in people over 80 years of age. 2

PD currently lacks a curative treatment; however, dopaminergic drugs have demonstrated significant efficacy in alleviating motor symptoms. Achieving optimal therapeutic outcomes relies on the crucial factor of patient adherence to the prescribed medication. World Health Organization (WHO) defines “adherence to (or compliance with) a medication” as “the extent to which a person's behavior —taking medication, following a diet, and/or executing lifestyle changes—corresponds with agreed recommendations from a health care provider” 3 . According to the WHO, a patient's behavior may be affected by the patients themselves, health system/health care, social/economic, therapy, and health conditions. Studies with a high level of evidence have shown that medication adherence in PD tends to be suboptimal. A Slovak study evaluating 219 PD patients found a high degree of adherence to treatment in only 52% of patients. 4 Grosset et al., 5 in a study carried out at Glasgow General Hospital in 2005, showed that complete adherence was observed in less than 3% in patients taking multiple doses of medications. A European multicenter study (involving 5 countries) showed that only 24.4% of the antiparkinsonian drugs doses were taken in the time interval considered correct. 6 However, it was not clear what impacted medication adherence in PD patients.

Socioeconomic factors impact on medication adherence is well established for some chronic diseases, such as hypertension and diabetes mellitus. 7 In hypertension, for example, some of the barriers to adherence were insufficient social support, the high cost of medications, and the patient's difficulty in understanding medical instructions. 8 The adoption of drug cost coverage policies can increase the adherence rate of benefited patients with chronic diseases. 9 If we consider other chronic neurologic diseases than PD, a study carried out in the USA showed that socioeconomic may impact medication adherence in dementia patients. 10 However, there is no large cohort or systematic review analyzing how these socioeconomic factors impact medication adherence among people with PD (PwPD). Understanding this global health problem is especially important for developing countries. In São Paulo, Brazil, for example, the average annual cost related to PD per patient is US$5,853.50, and this value can even be higher in the more advanced stages of the disease. 11 Of this, 25% of the total expenses correspond to the cost of the medication itself. 11 This value becomes even more expressive when compared with the Brazilian annual gross minimum wage, which in 2023, was approximately US$3,170.00. Furthermore, there is evidence that 32.2% of people over 25 years of age in Brazil have incomplete primary education and 6.4% have no formal education. 12 Considering that poverty, low level of education, and other socioeconomic factors, can negatively impact medication adherence in chronic diseases, our hypothesis is that this also may occur in PD. We performed a preliminary search of PROSPERO, Medline, the Cochrane Database of Systematic Reviews, and Joanna Briggs (JBI) Evidence Synthesis and observed that no current or in-progress scoping reviews on the topic were identified. Our purpose is to explore whether socioeconomic factors impact medication adherence among PwPD.

## METHODS

On March 5, 2022, we conducted a systematic scoping review to locate studies published in three databases to answer the following review question: how do socioeconomic factors impact medication adherence among PwPD?

### Eligibility criteria

To address the specific question, we examined peer-reviewed original studies, of quantitative, qualitative, and mixed-methods approach, that investigated and described the influence of socioeconomic factors on medication adherence in PwPD.

We did not put any year or language restrictions and included all studies published until March 5, 2022. We excluded study protocols, conference proceedings, editorials, and letters to the editors. For identified reviews, we searched the primary studies and included those meeting the eligibility criteria of our study.

### Search strategy and study selection

We made the search for related publications on PD medication adherence as follows:

- In February 2022, we initially carried out a Medline (PubMed) search to identify studies on the topic using the words contained in the titles and abstracts of relevant studies, and the index terms used to describe the studies, with the intention to develop a full search strategy.
- In March 2022 we expanded the search by including all identified keywords and index terms found in step one and adapted them for each database, including Embase (Elsevier) and LILACS (Virtual Health Library Portal) ( **Supplementary Material** - https://www.arquivosdeneuropsiquiatria.org/wp-content/uploads/2023/11/ANP-2023.0174-Supplementary-Material.docx ). Following the search, we identified all studies and uploaded them into Rayyan ( https://www.rayyan.ai/ ), for screening of abstracts and titles and for taking duplicate citations out. After that, two of us (GG and HBF), independently, screened titles and abstracts in accordance with the pre-established inclusion and exclusion criteria for the review. Studies potentially considered relevant were retrieved in full, and their citation details imported into the reference manager Mendeley v.1.19.4 (Mendeley Ltd., Elsevier, Netherlands). The same two reviewers assessed the full text of selected citations and evaluated them taking into consideration the adopted inclusion criteria, recording, and reporting in this review all the reasons for the exclusion. Disagreements appearing between the reviewers during the selection process were solved through discussion.
- In August 2022, we reviewed all the selected studies. The results of the search, study selection, and inclusion process were reported in full in the final scoping review and presented in a Preferred Reporting Items for Systematic Reviews and Meta-Analyses extension for scoping reviews (PRISMA-ScR) flow diagram. 13

### Data extraction, analysis, and presentation

We extracted the data from included studies using a Microsoft Excel spreadsheet for Mac, version 16.66.1 according to the variables to be investigated in our review: study characteristics, population, and socioeconomic attributes. We classified the socioeconomic factors according to eight attributes 3 7 14 :

- Educational level,
- Knowledge about the disease,
- Income,
- Cost of medication,
- Drug subsidy (meaning presence of subsidies in the cost of medication),
- Employability, and
- Ethnicity.

We considered ethnicity as a socioeconomic factor, since in many countries, blacks, indigenous, and immigrants have a lower level of income and employment as compared to the rest of the population. For quantitative descriptive design studies, we extracted the figures and percentages of all attributes, regardless of their prevalence, but we included only those cited by 10% of the sample. For quantitative analytical design studies, we extracted the linear or logistic regression data statistics including only those with significant inferential relationship with the phenomena of interest in a way that answered the review question. For qualitative studies, we extracted and categorized all the attributes identified in the qualitative data to generate a single comprehensive set of categories that could be used in an evidence-based practice. Data are presented in diagrammatic and tabular form following the scope review guidelines.

## RESULTS

**Figure 1:**
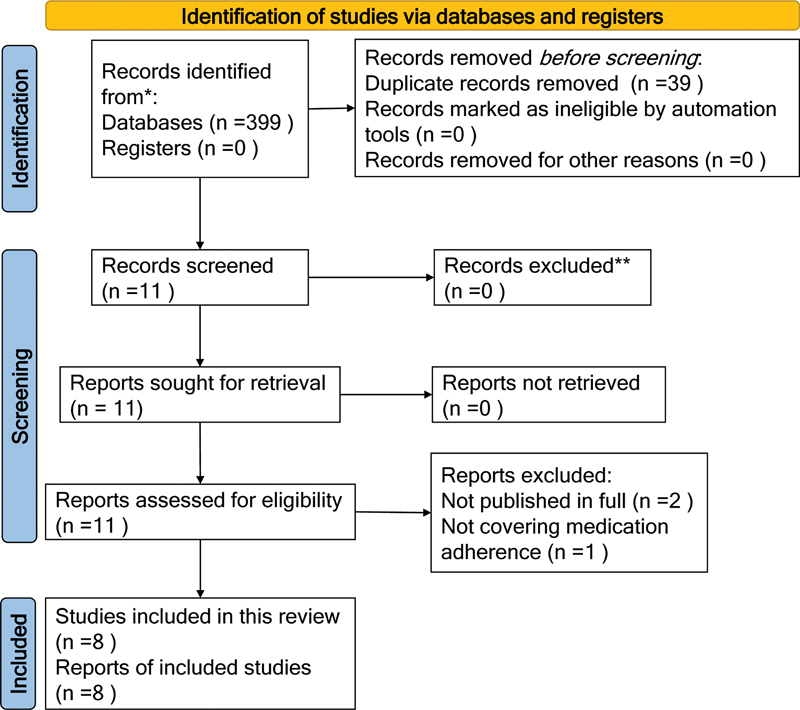
Search results and study selection and inclusion process. Source: Page MJ, McKenzie JE, Bossuyt PM, Boutron I, Hoffmann TC, Mulrow CD, et al. The PRISMA 2020 statement: an updated guideline for reporting systematic reviews. BMJ 2021;372:n71. doi: 10.1136/bmj.n71.

We initially screened 399 studies. Of these, 49 duplicates were removed, and 339 were excluded for not meeting the eligibility criteria. We identified 11 studies as being potentially relevant to the review and retrieved them for full-text assessment. Of these, 3 were excluded (2 for not having been published in full 15 16 and 1 for not covering medication adherence 17 ( Figure 1 ).

Of the 8 selected studies, seven were of quantitative design, being three of them cross-sectional 18 19 20 , three retrospective, 21 22 23 and one observational. 24 One study had a qualitative descriptive design. 25

The Table 1 summarizes the findings of the selected studies. We identified studies evaluating the socioeconomic factors impacting medication adherence among PwPD in 10 countries and 12,559 patients. The selected studies came from North America (10,951 patients), 21 22 23 25 followed by Europe, being one study from Spain with 450 patients 18 and one from Germany with 226 patients. 24 We identified one multicentric study coming from South America (Mexico, Argentina, Ecuador, Colombia, Chile, and Peru) with 800 patients, 19 and one study from Asia (India) with 134 patients. 20 We could observe that all eight pre-defined attributes negatively impact medication adherence among PwPD.

**Table 1:** Characteristics and findings of the studies included in the review

Autor, year, Country	Socioeconomic factors assessed	Design, medication adherence assessment	Sample size (N)	Mean age of subjects, and PD progression (years)	Socioeconomic factors with significant impact on medication adherence (MA)
Vallderiola et al, 2011, Spain	1. Disease-related knowledge 2. Income	Cross-sectional Multicentric MGT	450	Age: 70.2 ± 9.6 PD: 5.7 ± 5.4	Higher knowledge → Higher MA Higher wages → Higher MA
Wei et al, 2013, United States	1. Ethnicity 2. Drug subsidy	Retrospective MPR	7583	Age: < 65: 6,4% 65–74: 27,4% 75–84: 46% ≥ 85: 20,2% * PD: Not reported	Non-white participants have lower MA Subsidy related to lower adherence
Reynolds et al, 2020, United States	1. Income 2. Level of Education 3. Ethnicity 4. Costs of medications	Retrospective MPR	3130	Age: 70.6 ± 10.8 PD: Not reported	Higher income → Higher MA Higher costs of medications → Lower MA
Parashos et al, 2004, United States	1. Costs of medications	Retrospective Rate of discontinuation of medications	222	Age: 68.6 ± 10.3 PD: 9.4 ± 6.0	Higher costs of medications → Lower MA
Shin et al, 2015, United States	1. Costs of medications 2. Disease-related knowledge	Qualitative Interviews	16	Age: 68.1 (range: 53 to 82 years)** PD: From 6 months to 14 years**	Higher costs of medications → Lower MA Higher Knowledge about Parkinsonian Drugs → Higher MA
Mendorf et al, 2020, Germany	1. Disease-related knowledge 2. Level of education	Observational SAMS	226	Age: 71.1 ± 7.9 PD: 9.4 ± 6.9	Lower level of education → Lower MA
Castro et al, 2021, Mexico, Argentina, Ecuador, Colombia, Chile and Peru	1. Level of education 2. Employability 3. Drug subsidy	Cross-sectional Multicentric SMAQ	800	Age: 65.08 ± 11.37 PD: 8.06 ± 5.78	Lower level of education → Lower MA Subsidy related to lower MA
Aggarwal et al, 2021, Índia	1. Employability 2. Level of Education 3. Income	Cross-sectional MMAS-8	132	Age: 61.1 ± 10.3 PD: 4.5 ± 3.3	No differences between variables evaluated

Abbreviations: MGT, Scores of Morisky-Green Test; MPR, Medication Possession Ratio; PD, Parkinson's disease; SAMS, Stendal Adherece with Medication Score; SMAQ, Simplified Medication Adherence Questionaire; 8-item MMAS-8, Morisky Medication Adherence Scale.

Vallderiola et al. observed that having a higher income (incomplete numerical data) and having greater disease-related knowledge were associated with good medication adherence (62.8% of adherence among those who had a high level of disease-related knowledge vs. 51.02% adherence among those who had a low; p = 0.04). 18 The South American study related medication adherence to sociodemographic and clinical factors by the Simplified Medication Adherence Questionnaire (SMAQ) 19 and found that less adherent patients had fewer years of education (9.1 - 10.0 years for non-adherent and 10.9 - 11.9 years for adherent, CI 95%) and higher unemployment rate (79.4% of unemployed non-adherent patients vs 70.0% of unemployed adhered patients, p = 0.002). Furthermore, a lower level of adherence was observed in patients who received their medications without paying for them. The Indian study found that suboptimal medication adherence was directly related to irregular frequency of appointments (a patient-related factor), and the presence of side effects and depression (both disease-related factors). 20 This study was not able to find a statistically significant correlation between low adherence and the educational level (no numerical data presented), social class (comparison of adherence between upper, middle, and lower classes), and unemployment (no numerical data presented). 20 It should be noted that only 2.2% of the participants of the study were from low socioeconomic status. 20

Two retrospective studies showed that lower prices charged for dopamine agonists and drug costs partially covered by the US government (Part D Low Income Subsidy patients) were related to higher rates of adherence. 21 22 Reynolds et al. 22 evaluated the relationship between price and adherence to treatment of two dopamine agonists: ropinirole and pramipexole. They observed that, between 2004 and 2007, when drugs had the same price, the Medication Possession Ratio (MPR), used to calculate an individual's adherence to treatment, showed a very small difference between them (0.003 and 0.0053, respectively). However, when pramipexole became more expensive than ropinirole, the differences in the MPR became greater (between 0.03 and 0.09 respectively), with a higher adherence rate for those taking ropinirole. Furthermore, the authors noted that patients with household incomes more than 100,000 dollars annually had higher adherence rates than those earning less than 40,000 dollars. The study also showed a higher adherence in Caucasian as compared to non-Caucasian patients. 22

Wei et al. 21 observed that white individuals had higher adherence to medication as compared to non-white people (95% CI - 0.81–0.98). This difference became even more evident when black and white individuals were compared.

Another retrospective study showed that cost was the main reason for discontinuation of treatment in patients taking entacapone (one of the antiparkinsonian drugs). 23 Other reasons for discontinuing entacapone were a lack of efficacy (46%) (a therapy-related factor), worsening of PD symptoms (28.2%), cognitive dysfunction (20.2%), dyskinesia (16.9%), nausea (10.5%), diarrhea (8.9%) (all disease-related factors). 23

In a qualitative study, evaluating the difficulties and challenges to adherence to treatment, five of the 16 participants reported that the cost of medications, even those with partial coverage by health insurance companies, was a real concern. 25 A group of patients also reported that seeking knowledge about antiparkinsonian drugs also helped them to understand the adverse effects of medications, and consequently to adhere to treatment. 25

An observational study also correlated low adherence with a low level of knowledge about the prescribed drug, which was directly associated with the low educational level (patients who did not attend a school or who did not complete secondary education) (p = 0.002)24. For the nonadherent patients, 32.5% reported that lack of knowledge about the prescribed drug, such as the exact name, dosages, and the reasons for treatment, was the main reason. 24

## DISCUSSION

This study shows that socioeconomic factors have a high impact on medication adherence among PwPD. One of the studies of this review found a relationship between non-white ethnicity and lower adherence to PD treatment. 21 The same pattern of medication adherence has been found in other chronic diseases. One study from the USA observed that the adherence to medical treatment for diabetes mellitus, hypertension, and dyslipidemia was, on average, 7.5% lower in black and Hispanic as compared to white individuals. 26 Other studies also showed that African-American individuals present lower adherence to treatment in diseases such as dyslipidemia and systemic lupus erythematosus. 27 28 The study of Wei et al., 21 despite being carried out in the USA, reflects a reality that is very frequently present in several countries, where ethnic inequality in accessing health services is a manifestation of historical inequalities. Afro-descendant and Hispanic individuals in the USA are often associated with poor economic conditions, greater social vulnerability, and more severe health problems. 29 This is not different from what is seen in developing countries like Brazil in the recent years of the COVID-19 pandemics. 30

Reynolds et al. 22 observed no statistically significant relationship between ethnicity and adherence to treatment. The low rate of non-white PD patients participating in the study (86 Asian, 195 black, and 312 Hispanic, as compared to 2,351 white individuals) may explain the finding. In the same study, analyzing patients with dementia and neuropathy, again, statistically significant higher adherence was found in white patients as compared to non-white patients. 19 Lower family income was also considered a predictor of lower adherence when compared to higher family income, and this becomes evident in the study by Vallderiola et al. 18 Other studies carried out in developing countries reinforce this thesis. In Kenya, antiparkinsonian drugs are not regularly available in public and private pharmacies (levodopa, for instance, is available only in 50% of public pharmacies), and the medications are not affordable for most of the population. 31 In Gujarat, a rural city in India, patients spend an average of US$ 123 annually for PD treatment, which corresponds to 6.8% of their annual gross income. 32 A Chilean study showed that PD patients who received higher salaries had access to high-cost drug associations, such as levodopa and dopaminergic agonists, while poorer patients had only access to levodopa. 17 Another study comparing PD treatment in Ghana and Italy observed that the initiation of treatment with levodopa in Ghana is much later than in Italy since levodopa is not easily available in the African country. 33 Additionally, the higher costs of dopamine agonists and COMT inhibitors make amantadine and anticholinergics a first-line treatment in Ghana. 33 Accessibility to antiparkinsonian drugs remains a huge problem in many African countries. 34 5966

Unemployment is directly related to a worse socio-economic condition of individuals and is also seen as an obstacle to long-term therapy adherence in diseases such as schizophrenia and kidney transplantation. 35 36 As well as lower wages, unemployment is one of the causes of lower income, which would explain the difficulty in maintaining therapy among PwPD, as pointed out by Castro et al. 19 Partial covering costs of antiparkinsonian drugs make the medication adherence rate higher. 21 22 23 25 A systematic review of diabetes showed that the higher the cost of the medication the lower the adherence to treatment. 37 In the USA, a higher subsidy given to low-income patients increases the adherence of beneficiaries and eventually generates lower costs for the health plan. 38 In the same way, low-income patients who do not receive subsidies have very low adherence. 39

Contrary to what other studies have shown, 37 39 40 Castro et al. 21 found that free access to antiparkinsonian drugs decreased the rate of adherence. The authors point out that this finding should be analyzed carefully, and they attribute it to an educational issue. They consider that, despite receiving the drugs free of charge, patients may not use them correctly for not being adequately informed about the therapeutic regimen. Moreover, the authors indicate that lower educational levels and less involvement in disease control, despite having free access to medication, could be a plausible explanation. 21

Parashos et al. 23 observed that only 4% of the patients had the medication cost as a justification for discontinuing treatment. We should consider that this study was carried out at a private institution, where patients would likely have little difficulty purchasing medications. Another possible explanation is that some individuals may be less confident in the effectiveness of nonpaid medications and may not use them properly. 23

The level of education is critical for good adherence to PD treatment. 24 Patients that have a good knowledge of their disease and those who understand how meaningful is to keep regular treatment usually have more years of education. 41 42 PD patients who receive extra information about their disease may also improve adherence to treatment. 43 We believe that the level of education is directly related to earning higher wages and the combination of these two factors may improve the adherence to PD treatment.

### Study limitations

We identified some limitations in this review. First, we identified studies only in four continents, and few non-North American or non-European studies, and this may have impacted the findings since socioeconomic factors are directly related to culture. Second, the variety of outcome measures was quite diverse for a meta-analysis to be undertaken and data from the quantitative studies of this review had to be presented in a descriptive format. Third, the number of patients evaluated varied from small samples to large cohorts of meta-analysis studies. Fourth, the lack of qualitative studies may have particularly influenced which and how socioeconomic attributes impact medication adherence among PwPD. The findings of our review were restricted to the components of the rating scales used in the quantitative studies. Studies with a qualitative approach by nature allow unlimited exploration of the phenomenon of interest, giving voice to the individual experiencing the problem.

In conclusion, we could observe that socioeconomic factors negatively impact medication adherence among PwPD. This review provides the basis for developing patient and population-based interventions to improve medication adherence. Some of the factors cannot be modified by the health team/system (such as income), while others can be addressed in the individualized approach to health (such as knowledge about the disease).
